# Application of Disulfiram and its Metabolites in Treatment of Inflammatory Disorders

**DOI:** 10.3389/fphar.2021.795078

**Published:** 2022-02-02

**Authors:** Wenyi Guo, Shihong Chen, Chengqing Li, Jianwei Xu, Lei Wang

**Affiliations:** ^1^ Department of Pancreatic Surgery, General Surgery, Qilu Hospital, Cheeloo College of Medicine, Shandong University, Jinan, China; ^2^ Department of Pancreatic Surgery, General Surgery, Qilu Hospital, Shandong University, China

**Keywords:** disulfiram, inflammation, pyroptosis, NLRP3, GSDMD, ROS

## Abstract

Disulfiram has been used clinically for decades as an anti-alcoholic drug. Recently, several studies have demonstrated the anti-inflammatory effects of disulfiram and its metabolism, which can alleviate the progression of inflammation *in vivo* and *in vitro*. In the current study, we summarize the anti-inflammatory mechanisms of disulfiram and its metabolism, including inhibition of pyroptosis by either covalently modifying gasdermin D or inactivating nod-like receptor protein 3 inflammasome, dual effects of intracellular reactive oxygen species production, and inhibition of angiogenesis. Furthermore, we review the potential application of disulfiram and its metabolism in treatment of inflammatory disorders, such as inflammatory bowel disease, inflammatory injury of kidney and liver, type 2 diabetes mellitus, sepsis, uveitis, and osteoarthritis.

## Introduction

Inflammation is an adaptive response of the body to harmful stimuli, which has a protective effect on the body under normal circumstances (Medzhitov, 2008). However, if the protection is disordered, the inflammatory response will be excessive, harmful effects are produced and then develop to diseases (Medzhitov, 2008). Inflammation is involved in the onset of many diseases, and controlling the progression of inflammation has become a key task in the treatment of these diseases. There are many inflammatory diseases and the treatment options vary a lot, however, the effects are uneven. Especially for some severe diseases such as severe acute pancreatitis and severe pneumonia, there is still a certain mortality rate even after systematic treatment (De Pascale et al., 2012; Zerem, 2014; Gotts and Matthay, 2016; Rello et al., 2017), so it is extremely necessary to find medicines to treat inflammatory diseases.

Disulfiram (DSF), a drug used to treat alcoholics, has been used for more than 50 years and is well tolerated by patients (Fuller et al., 1986). DSF is metabolized in the blood into diethyldithiocarbamate (DDC) or the DDC copper complex, and the metabolites of DSF after ingestion are proportional to the intake doses (Johansson, 1992). DDC is further metabolized to the corresponding sulphoxide and sulphone metabolites, which are inhibitors of ALDH 1 and ALDH 2 (Johansson, 1992; Zhang et al., 2013), leading to the accumulation of acetaldehyde when ethanol is ingested (Schroeder et al., 2010), in turn causes a series of uncomfortable reactions such as nausea, vomit, dizziness, and headache, so as to have an effect in quitting alcohol. In addition, recent studies show that DSF has anti-cancer effects that have been identified and part of their mechanisms are described in detail (Sreerama and Sladek, 1993; Skrott et al., 2017; Wang et al., 2020). Besides, many researchers found its anti-inflammatory effects and the mechanism was also revealed to some extent recently. Since Gunasekaran et al. reported that DSF could prevent inflammatory edema after spinal cord injury in 1985 (Gunasekaran et al., 1985), DSF and its metabolites have been found to be effective in a large number of inflammatory diseases. In this article, we briefly summarized the mechanism of DSF and its metabolites in the treatment of inflammatory diseases.

## Anti-Inflammatory Mechanisms of Disulfiram

### Effects on Pyroptosis

#### What is Pyroptosis

Pyroptosis is a type of cell death, which was first discovered in 1992 and defined in 2001 (Zychlinsky et al., 1992; Cookson and Brennan, 2001), and many signaling pathways are involved in pyroptosis (Fink and Cookson, 2005). Change in cellular morphology is the formation of cell membrane holes, resulting in cell swelling and rupture, then the cell contents such as inflammatory factors are released to stimulate inflammatory responses (Jorgensen and Miao, 2015; Galluzzi et al., 2018). Pyroptosis can be classified into canonical inflammation pathway mediated by caspase-1 and non-canonical inflammation pathway mediated by caspase-4/5/11 (Jorgensen and Miao, 2015). Many inflammasomes are involved in pyroptosis, such as nod-like receptor protein 3 (NLRP3), absent in melanoma 2 (AIM2), NLRP1 and so on (Vande Walle and Lamkanfi, 2016). These inflammasomes can activate caspases that cut gasdermins into C-terminal and N-terminal gasdermins. The N-terminal gasdermins can perforate the cell membrane to cause cell swelling and rupture, so that release of inflammatory factors such as IL-1β and IL-18 results in inflammation (Ding et al., 2016), and completion of the pyroptosis process.

#### The Direct Effect of Disulfiram on Gasdermin D (GSDMD)

GSDMD is a member of the gasdermin protein family and plays an important role in the pyroptosis process. After being cleaved to N-terminal and C-terminal GSDMD by caspase-1/4/5/11, the N-terminal fragment can be transferred to the plasma membrane and form a membrane pore (Sborgi et al., 2016). In turn, IL-1β, IL-18 and other inflammatory mediators are released through this pore (Shi et al., 2015). Hu JJ et al. demonstrated in mouse experiment that DSF can inhibit the release of IL-1β without affecting other proteins such as caspase-1 and pro-IL-1β, thus confirming that DSF could inhibit pyroptosis by inhibiting GSDMD, and further confirming that DSF could covalently modify cys192 of GSDMD, rather than other gasdermins, inhibiting plasma membrane pore formation and the pyroptosis process (Hu et al., 2020). Furthermore, DSF can reduce the release of inflammatory mediators such as tumor necrosis factor (TNF) and IL-6 through the GSDMD pathway (Hu et al., 2020), thus reducing the inflammatory response to some extent. And it can be inferred that all pyroptosis pathways ending in GSDMD will be affected by DSF.

#### The Effect of Disulfiram on the Nod-Like Receptor Protein 3 Inflammasome and Indirect Effect on GSDMD

The NLRP3 inflammasome is one of the pyroptosis-associated inflammasomes leading to activation of caspase-1 which determines IL-1β and IL-18 maturation and release, contributes to pyroptosis (Jo et al., 2016). Deng et al. found that DSF could inhibit NLRP3, therefore inhibiting the release of IL-1β and the occurrence of cell pyroptosis in mouse J774A.1 and human THP-1 macrophage cell lines (Deng et al., 2020).

The activation of NLRP3 due to lysosomal destruction is also an important process during pyroptosis (Chen et al., 2015). Destabilization of the lysosomal membrane can lead to the release of cathepsin B, which activates the NLRP3 inflammasome (Newman et al., 2009; Jin and Flavell, 2010; Lamkanfi and Dixit, 2012), inducing the pyroptosis process. DSF can protect lysosomal membrane, reduce cathepsin B release therefore alleviating the inflammatory response (Deng et al., 2020).

### The Dual Effects of Disulfiram on Reactive Oxygen Species

Reactive oxygen species (ROS), which play an important role in the development of many inflammatory diseases, are produced from the NADPH oxidase or the mitochondrial respiratory chain in the process of aerobic metabolism of organisms (Mittal et al., 2014; Blaser et al., 2016). A large number of literatures have confirmed that the production of ROS is one of the key elements of NLRP3 activation (Tschopp and Schroder, 2010; Bauernfeind et al., 2011), activation of NLRP3 can be blocked when using ROS inhibitors (Zhou et al., 2011). Recently, researchers have discovered that LPS/nigericin-induced mitochondrial ROS did not decrease after adding DSF, indicating that DSF can reduce intracellular ROS production by reducing the source of NADPH oxidase (Deng et al., 2020). Furthermore, researchers found that DSF can inhibit NLRP3-dependent IL-1β secretion (Deng et al., 2020), so it can be inferred that DSF can inhibit NLRP3 activity by reducing ROS production.

### Disulfiram Inhibits Inflammation by Inhibiting Angiogenesis

Angiogenesis is an important stage in the inflammatory process, which is a key mechanism for leukocyte cells to enter the inflammation site through the vascular endothelium (Szekanecz and Koch, 2007). Angiogenic factors such as vascular endothelial growth factor (VEGF) and TNF-α can promote vessel formation (Leibovich et al., 1987; Marikovsky et al., 2003). DSF reduces TNF-α production and dose-dependently reduces the production of VEGF, thereby reducing angiogenesis and inflammation (Marikovsky et al., 2003). Another study has shown that DSF can reduce VEGF generation and inhibit angiogenesis *in vivo* by acting on the EGFR/Src/VEGF pathway, and this effect can be enhanced by the combination of copper (Li et al., 2015). Furthermore, DSF can cause ROS accumulation, induce intracellular oxidative stress, and cause endothelial cell growth arrest (Marikovsky et al., 2002), thus inhibiting angiogenesis.

## Disulfiram and Inflammatory Disorders

### The Therapeutic Effect of Disulfiram on Inflammatory Bowel Disease

Inflammatory bowel disease (IBD) is a chronic intestinal inflammatory disease of unknown etiology, including Crohn’s disease and Ulcerative Colitis (Moazzami et al., 2019), and has a certain risk of cancer (Bernstein et al., 2001). Clinically, abdominal pain and diarrhea are the main manifestations, which seriously affect the quality of life of patients (Sawczenko and Sandhu, 2003; Gupta et al., 2008). Oxygen free radicals play a role in tissue damage in the pathogenesis of colitis (Parks et al., 1983). Acetaldehyde dehydrogenase is responsible for the main source of ROS in colon (Sharon and Stenson, 1984), and the activity of ROS in tissues can be reflected in the level of molondialdehyde (MDA) when membrane lipids are damaged (Ohkawa et al., 1979). DSF can reduce MDA levels in the colonic tissues of rats with colitis (Bilsel et al., 2002). This may be due to the fact that DSF is the inhibitor of acetaldehyde dehydrogenase (Sawczenko and Sandhu, 2003; Gupta et al., 2008), however, the exact mechanism is still unclear.

### Disulfiram for the Treatment of Inflammatory Liver Diseases

Non-alcoholic fatty liver disease (NAFLD) is a kind of disease which is characterized by excessive accumulation of lipids in liver cells, and is the most common chronic liver disease, which can be divided into simple steatosis and nonalcoholic steatohepatitis (NASH) (Haas et al., 2016; Younossi et al., 2016; Lau et al., 2017). NASH manifests itself as steatosis and inflammatory damage to the hepatocyte. NAFLD can develop into liver cirrhosis and even liver cancer. Rats receiving methionine and choline deficient (MCD) diet can develop NASH (Van Herck et al., 2017; Liu et al., 2018), with fat accumulation, oxidative stress, and inflammation and fibrosis in liver cells (Hebbard and George, 2011; Ibrahim et al., 2016; Lau et al., 2017). It manifests itself mainly as a reduction in serum cholesterol (CHO) and high-density lipoprotein (HDL) levels and an increase in alanine transaminase (ALT) and endoplasmic reticulum stress and an up-regulation of cytochrome P450 2E1 in mouse liver, which can produce superoxide anion radicals (Leung and Nieto, 2013). These manifestations can be inhibited by the treatment with the diethyldithiocarbamate (DDC), the DSF metabolites (Liu et al., 2018). What’s more, DDC treatment can improve the liver function damage caused by MCD diet (Liu et al., 2018). Besides, the use of tetraethylthiuram DSF (TDSF) in animals on the MCD diet resulted in a significant reduction in inflammatory cell infiltration in the liver (Schwartz et al., 2013).

Alcoholic liver disease is a chronic liver disease caused by long-term heavy alcohol consumption, which can cause alcoholic hepatitis (Hines and Wheeler, 2004; Jerrells et al., 2007; Gao et al., 2019), and may develop into liver cancer (Altamirano and Bataller, 2011). Acetaldehyde levels increased in ALDH2 (-) mice when exposed to alcohol, and hepatitis was attenuated in ALDH2 (-) mice compared to wild-type mice (Hines and Wheeler, 2004; Jerrells et al., 2007; Gao et al., 2019). It can be inferred that DSF, as an ALDH inhibitor, can reduce hepatitis caused by alcohol use.

Mice on MCD diet developed liver fibrosis and collagen deposition can be observed around the central lobular vein. By reducing the aggregation of hepatic stellate cells (HSC) and myofibroblasts, DDC significantly reduced liver fibrosis induced by MCD diet (Hines and Wheeler, 2004; Jerrells et al., 2007; Gao et al., 2019).

### Disulfiram for Inflammatory Kidney Diseases

Renal fibrosis is the pathological response of the kidney to a variety of pathogenic factors such as inflammation and ischemia. When the kidney is stimulated by injury, the inflammatory pathway is activated and further activates pro-fibrotic cells (Black et al., 2019). NLRP3 plays an important role in the activation of renal fibrosis (Granata et al., 2015; Zhang and Wang, 2019). In the mouse model with NLRP3 knockout diabetic nephropathy, renal inflammation and fibrosis can be partially suppressed, suggesting that the pro-inflammatory effect of the NLRP3 inflammasome may promote renal fibrosis (Wu et al., 2018). Unilateral ureteral obstruction (UUO) is an experimental model of kidney injury and can cause renal fibrosis. After using DSF on UUO rats, the expression of IL-1β, IL-6, IL-18 and TNF-α in the peripheral blood and kidney tissues of the rats decrease significantly, and the degree of reduction was negatively correlated with the drug dose. What’s more, the use of DSF reduces the expression of GSDMD, and DSF could downregulate the level of α-SMA and upregulate the level of E-cadherin in renal tissues. These findings indicate that DSF can ameliorate renal fibrosis of UUO rats by inhibiting pyroptosis and other pathways (Zhang et al., 2021). Other studies have shown that DSF can reduce cisplatin-induced acute renal toxicity in rats by decreasing oxidative stress and inflammation (Khairnar et al., 2020).

### Disulfiram for Sepsis

Sepsis is a severe systemic inflammatory response following infection (Heumann et al., 1998), with a mortality rate of about 25% when no complication occurs or 80% when accompanied with multiple organ failure (Galley, 2011). Lipopolysaccharide (LPS) in the cell wall of Gram-negative bacteria is one of the stimulating factors that can cause sepsis (Heumann et al., 1998). LPS can induce sepsis in mice, while caspase-11 (-) mice were not induced to sepsis, suggesting that the non-canonical inflammasome pathway dominates LPS-induced sepsis (Kayagaki et al., 2011). Hu JJ at al. reported that DSF can prolong the survival time of LPS-induced sepsis mice. By the combination of copper, the therapeutic effect of DSF was further strengthened. These results suggest that DSF can inhibit sepsis induced by the non-canonical pyroptosis pathway (Hu et al., 2020).

### Disulfiram for Type 2 Diabetes

Type 2 diabetes is one of the most common diseases in internal medicine and has a high incidence in middle-aged and elderly people. Its hyperglycemic characteristic can lead to a series of complications (Vijan, 2015; American Diabetes Association, 2020). The onset of type 2 diabetes is associated with decreased pancreatic beta cell function and insulin resistance (Nagai et al., 2009; Zheng et al., 2018). Studies have shown that the onset of type 2 diabetes is related to ROS: glycation and the consequent increase of ROS can inhibit the transcription of insulin genes in mouse β-cell–derived HIT-T15 cells (Matsuoka et al., 1997). Based on rat models of type 2 diabetes, researchers found that oral administration of DSF increased insulin levels, improved glucose tolerance and reduced blood glucose and cholesterol levels in diabetic rats (Nagai et al., 2009). Furthermore, researchers found that DSF and its derivatives can covalently bind to the C128 site to inhibit fructose-1,6-bisphosphatase (FBPase), an important rate-limiting enzyme in the process of gluconeogenesis, thereby reducing blood glucose output (Huang et al., 2020). However, more in-depth research is needed on the treatment of DSF for diabetes.

### Disulfiram for Uveitis

Uveitis is considered an inflammatory disease that occurs in the uvea, the retina, the blood vessels of the retina, and the vitreous, and can cause blindness (Nussenblatt, 1990). The main pathophysiological characteristics are infiltration of inflammatory cells and accumulation of inflammatory factors in aqueous humor (Mo et al., 1999). DSF can dose-dependently reduce inflammatory cells infiltration and protein concentration in aqueous humor of LPS-induced rat model of uveitis, and decrease the levels of inflammatory factors such as NO, TNF-α and PGE2 in aqueous humor (Kanai et al., 2010; Kanai et al., 2011). However, its mechanism needs to be further elucidated.

### Disulfiram for the Inflammatory Response of Chondrocytes

Osteoarthritis is a chronic bone and joint disease that is more likely to occur in middle-aged and elderly people (Lespasio et al., 2017). In severe cases, it can cause joint pain and interfere with normal movement (Huang et al., 2015; Lespasio et al., 2017). Degenerative changes in articular cartilage can result in osteoarthritis (Goldring and Berenbaum, 2015). In C28/I2 cells (human chondrocytes), LPS and ATP can induce inflammation and pyroptosis (Li et al., 2021). Co-treatment with disulfiram and glycyrrhizic acid promote the proliferation and alleviate pyroptosis in LPS and ATP stimulated C28/I2 chondrocytes, and can reduce the production of ROS. DSF, when used at high concentrations, shows little effect on cell proliferation and pyroptosis (Li et al., 2021). This may provide new ideas for the treatment of osteoarthritis.

## Prospective

Inflammation plays a central role in the pathogenesis of many diseases. Therefore, the treatment of inflammation plays an important role in the treatment of many diseases. DSF has been used clinically for decades as an alcohol-abuse drug and plays a role in the treatment of other diseases such as cataract, obesity, myelodysplastic syndrome, leukemia, acquired immunodeficiency syndrome, Alzheimer’s disease, etc. (Vassar et al., 1999; Nagai and Ito, 2014; Elliott et al., 2015; Hassani et al., 2018; Reinhardt et al., 2018; Meggyesy et al., 2020; Yang et al., 2020; Zha et al., 2021). DSF is one of the older drugs that have been found to have anticancer and anti-inflammatory effects, and the effects on inflammation manifest as follows: ① inhibition of pyroptosis; ② inhibition of ROS; ③ inhibition of angiogenesis. There are many kinds of inflammatory diseases, and theoretically patients with diseases related to the anti-inflammatory mechanism of DSF may benefit from DSF therapy. In addition, the DSF analogues and the compound of DSF and copper may have the same effect as DSF, and the use of DSF in combination with other drugs may enhance anti-inflammatory effects.

However, the application of DSF is still very limited. The half-life of DSF is 7.3 h in plasma (Johansson, 1992), which is metabolized into other substances soon after ingestion and its metabolism is complex in the human body. According to current reports, DSF has a wide range of treatments *in vivo* and *in vitro* experiments and seems to be a panacea. This phenomenon is related to DSF metabolites or the mechanism of action, and it is necessary to further elaborate the anti-inflammatory mechanism of DSF. The current researches on DSF are basically the studies of DSF itself, not its metabolites. However, there is currently no evidence of pharmacological activity of DSF as a molecular entity *in vivo* throughout all the medical or biomedical researches (even in the treatment of alcoholism), so the studies using disulfiram instead of its metabolites can’t explain what actually works *in vitro* or when animals are given oral or intraperitoneal injections of DSF. So many studies did not mention this issue, which is not rigorous and may exaggerate the effect of the drug; this could also explain the panacea illusion. Furthermore, the dosage of DSF *in vivo* and *in vitro* experiments far exceeds the recommended dose of the drug, which is 500 mg/day for humans (Brewer, 1984), so it is necessary to consider whether it can reach the effective concentration when applied to humans. Therefore, in order to truly study the effects of DSF more thoroughly, researches should use DSF metabolites (including DDC copper complex or those not mentioned in this review) *in vivo* and *in vitro* experiments, and strictly control their dosage. Finally, the current studies are all preclinical studies, so it is hard to determine whether there will be side effects and serious consequences in the future clinical applications. Therefore, after confirming the therapeutic effect of DSF on diseases, how to rationally apply the drug in clinical practice has also become a problem worth considering. With the deepening of research, more and more functions of DSF have been revealed. DSF is believed to play an increasingly important role in clinical practice in the future.
