# Supplementary figures and images for: Application of Disulfiram and its Metabolites in Treatment of Inflammatory Disorders
Source: Front Pharmacol. 2022 Feb 2;12:795078. doi: 10.3389/fphar.2021.795078 (PMC8848744; doi:10.3389/fphar.2021.795078)

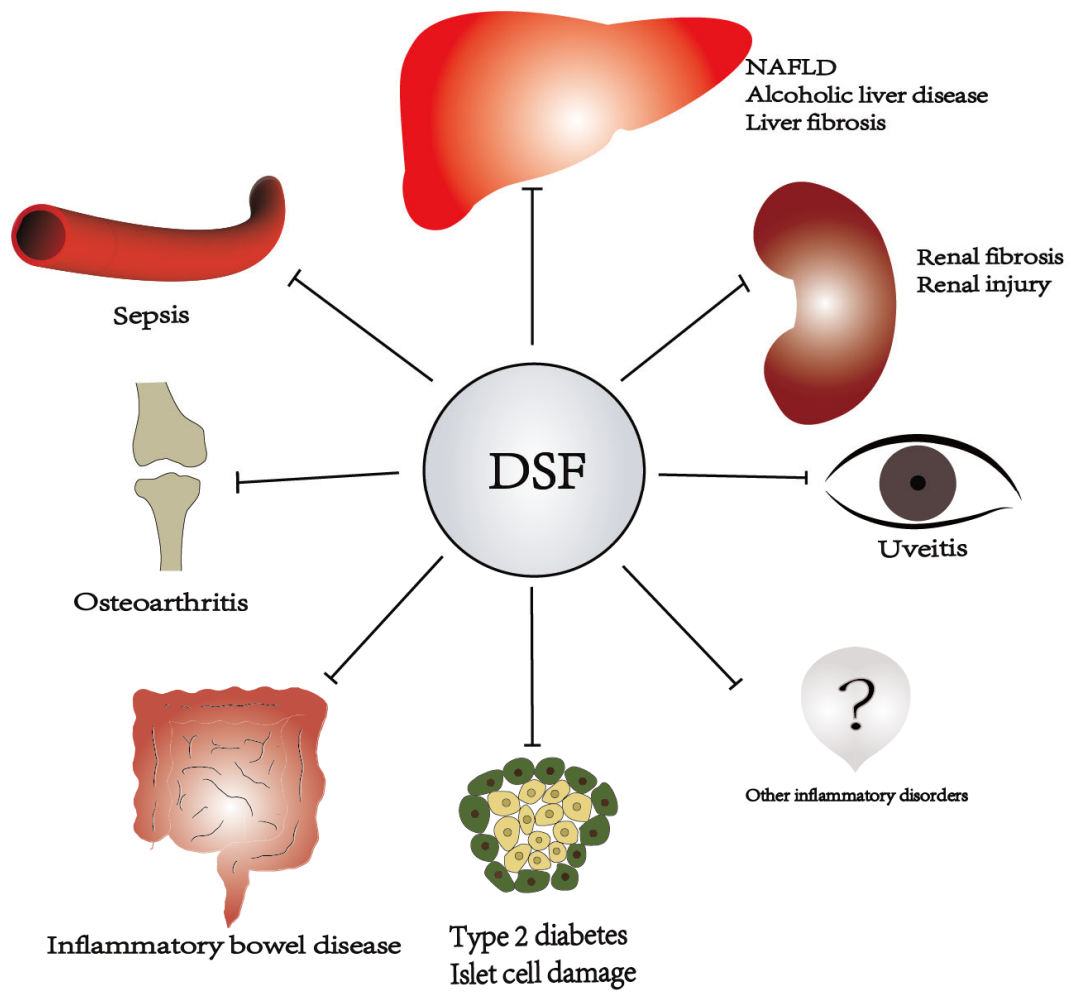

**Figure 2: DSF for inflammatory disorders**

Supplement: Supplementary file 1 [file Image2.pdf]

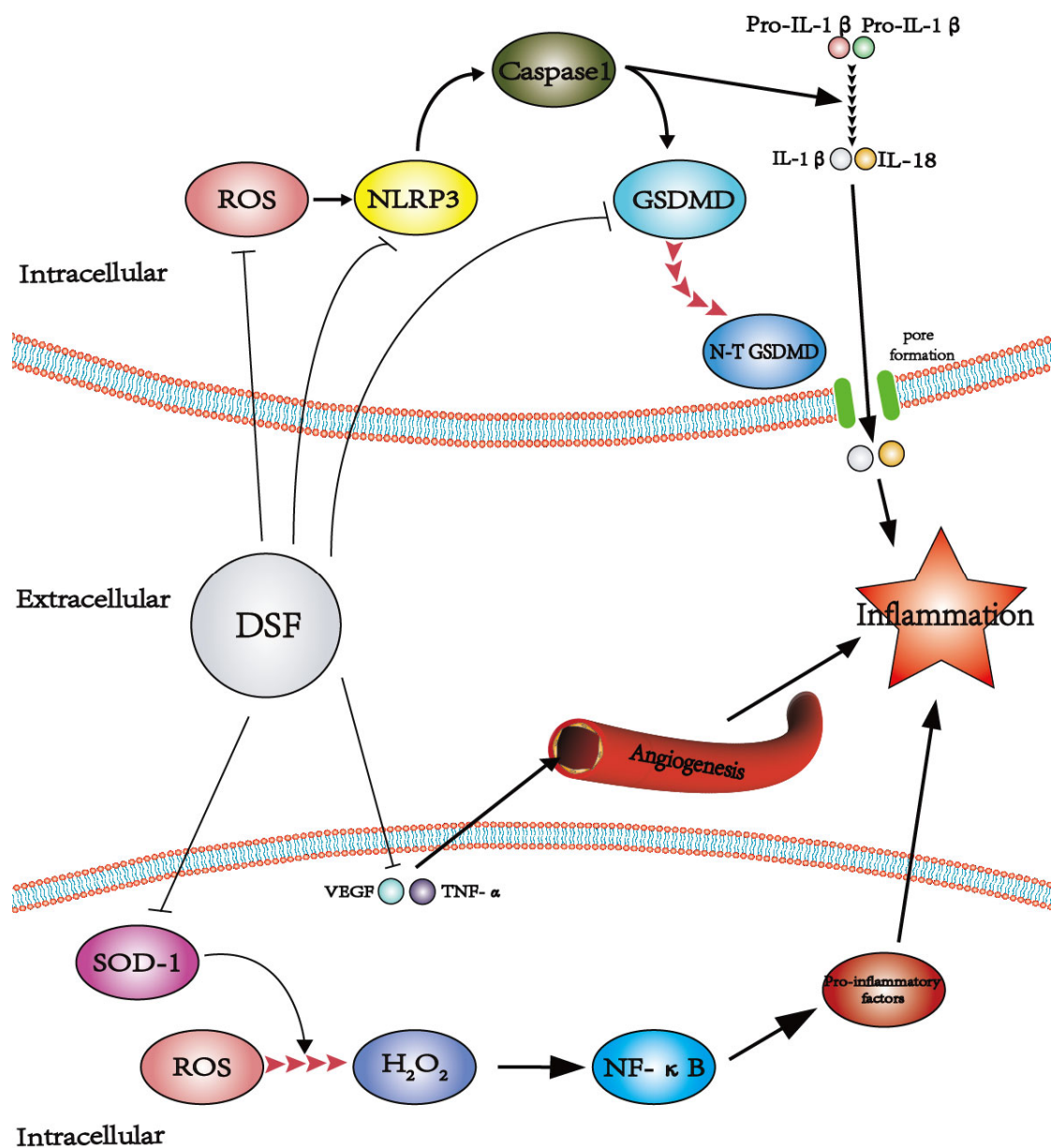

**Figure 1: Anti-inflammatory mechanism of DSF**

Supplement: Supplementary file 2 [file Image1.pdf]
